# ELOVL5-Mediated Long Chain Fatty Acid Elongation Contributes to Enzalutamide Resistance of Prostate Cancer

**DOI:** 10.3390/cancers13163957

**Published:** 2021-08-05

**Authors:** Huan Xu, Sangsang Li, Yi Sun, Lingfan Xu, Xin Hong, Zhong Wang, Hailiang Hu

**Affiliations:** 1 Department of Urology, Shanghai Ninth People’s Hospital, Shanghai 200011, China; 120094@sh9hospital.org.cn; 2 Department of Urology, Shanghai Changhai Hospital, Shanghai 200433, China; 3 Department of Biochemistry, School of Medicine, Southern University of Science and Technology, Shenzhen 518055, China; liss3@mail.sustech.edu.cn (S.L.); hongx@sustech.edu.cn (X.H.); 4 Department of Urology, Minimally Invasive Surgery Center, The First Affiliated Hospital of Guangzhou Medical University, Guangzhou 510120, China; sunyi@scu.edu.cn; 5 Guangdong Key Laboratory of Urology, Guangzhou 510120, China; 6 Department of Urology, The First Affiliated Hospital of Anhui Medical University, Hefei 230022, China; ayfyxlf@163.com

**Keywords:** enzalutamide resistance, neuroendocrine prostate cancer, fatty acid elongation, poly unsaturated fatty acid, lipid raft

## Abstract

**Simple Summary:**

The resistance mechanism of hormonal therapy has been a long-sought-after but not-yet-understood research topic in the prostate cancer (PCa) field. Here, we provide new mechanistic insights into how long-chain fatty acid contributes to enzalutamide resistance of prostate cancer. We demonstrated that ELOLV5-mediated polyunsaturated fatty acids (PUFAs) upregulation and the lipid raft-derived activation of AKT-mTOR pathway drives the therapy resistance and neuroendocrine differentiation (NED) of prostate cancer. Thus, ELOVL5 could be a potential candidate for therapeutically targeting the therapy-resistant NE-like PCa.

**Abstract:**

Prostate cancer (PCa) exhibits an elevated level of de novo lipogenesis that provides both energy and basic metabolites for its malignant development. Long-chain polyunsaturated fatty acids (PUFAs) are elongated and desaturated from palmitate but their effects on PCa progression remain largely unknown. Here, we showed that PUFAs were significantly upregulated by androgen deprivation therapy (ADT) and elevated in neuroendocrine (NE)-like PCa cells. The key enzyme of PUFA elongation, ELOVL5, was overexpressed in NE-like PCa cells as well. Furthermore, we demonstrated that knocking down ELOVL5 in enzalutamide resistant NE-like PCa cells diminished the neuroendocrine phenotypes and enzalutamide resistance, while overexpressing ELOVL5 augmented the enzalutamide resistance of PCa cells in vitro and in vivo. Mechanistically, ELOVL5-mediated PUFA elongation enhanced the lipid raft-associated AKT-mTOR signaling activation and therefore contributes to the enzalutamide resistance. These findings suggest that ELOLV5-mediated PUFA elongation may be a potential novel target for the treatment of enzalutamide resistant NE-like PCa.

## 1. Introduction

Prostate cancer (PCa) is the most common malignancy in men which is also the second leading cause of cancer deaths in the developed countries [1]. Hormone therapy, including the standard androgen deprivation therapy (ADT) and the second generation of anti-androgen drugs such as enzalutamide or abiraterone, is the most commonly used treatment for PCa [2,3]. However, castration resistant prostate cancer (CRPC) is inevitably developed and progressed in most PCa patients after hormonal therapy [4]. Neuroendocrine prostate cancer (NEPC) is a variant of CRPC with small cell neuroendocrine phenotype that is thought to be a cellular mechanism that mediates the hormonal therapy resistance, including enzalutamide resistance, and finally leads patients to death [5,6].

Prostate cancer incidence has been significantly increased in developing countries due to the wide use of the PSA (prostate specific antigen) screen and the increased prevalence of a Western diet [7]. Altered lipid metabolism is known as a hallmark of prostate cancer cells, especially the fatty acids’ biosynthesis and biological activities [8]. It has also been acknowledged that overexpression of several lipogenic enzymes is relative to prostate cancer tumorigenesis [8]. The blockade of de novo lipogenesis and lipid uptake can inhibit the growth of PCa cells [8]. PUFA (polyunsaturated fatty acid) has been demonstrated to have anti-inflammation and anti-cancer effects and Arachidonic acid (AA), a major type of PUFA, has been shown to be involved in prostate carcinogenesis [9,10]. Furthermore, very long chain PUFAs of the n-6 and n-3 series are key components of membrane phospholipids which are precursors of cell signaling and important for the development of drug resistance [10]. Lipid rafts are microdomains of plasma membrane enriched in cholesterol and sphinolipids, playing a key role in cellular signaling transduction [11]. N-3 PUFA has been shown to alter the lipid raft composition and increases the EGFR signaling [12]. Oncogenic ATK signaling can be activated by the changes of cell membrane contents, such as increased lipid rafts, and further contributes to hyperactivation of mTORC1 and mTORC2 [8], and as a consequence, the activation of the AKT-mTOR pathway can contribute to the enzalutamide resistance of prostate cancer [13]. Therefore, cell membrane architecture and its initiated signaling pathways are regulated by the PUFAs. Furthermore, fatty acid desaturation can determine the membrane fluidity and therefore lead the cells to become more migratory [14].

Fatty acid elongase-5 (ELOVL5) elongates γ-linolenic acid (18:3, n-6) to form dihomo-linolenic acid (20:3, n-6) that can be further transformed into arachidonic acid (20:4, n-6) by desaturase 1 (FADS1). ELOVL5 is one of seven ELOVL family members expressed in mammals [15,16]. Using malonyl CoA, NADPH, and fatty acyl-CoA as substrates, ELOVL5 can catalyze two-carbon additions to both PUFAs and MUFAs, such as the synthesis of C20 n-6 PUFAs (Arachidonic acid, 20:4, n-6) and C22 n-3 PUFAs (DHA, 22:6, n-3) as well as the n-7 class of MUFAs like cis-vaccenic acid (cis-VA, 18:1, n-7). Therefore, ELOVL5 is one of the most important enzymes in the fatty acid chain elongation, especially for the PUFA production. A most recent study showed that ELOVL5 is a critical and targetable fatty acid elongase in prostate cancer metastasis and its expression can be regulated by androgen receptor [17]. However, the effect of fatty acid elongation and ELOVL5 on neuroendocrine prostate cancer differentiation as well as hormone therapy resistance is still unknown. In our study, we aim to find the effect of fatty acid elongation on NEPC differentiation and hormone therapy resistance.

In this study, we found that PUFAs were significantly elevated in enzalutamide resistant NE-like PCa cells and its elongation enzyme ELOVL5 was overexpressed in NE-like PCa cells as well. Further, we demonstrated that ELOVL5-mediated PUFA elongation enhanced the lipid raft-associated AKT-mTOR signaling activation that may contribute to the enzalutamide resistance of PCa.

## 2. Materials and Methods

### 2.1. Cell Culture

LNCaP, C4-2, PC3 cells (ATCC, Manassas, VA, USA) were cultured in RPMI-1640 medium (Gibco, New York, NY, USA) supplemented with 10% fetal bovine serum (FBS; Corning, Corning, NY, USA) or 10% delipidized Fetal Bovine Serum fetal bovine serum (Sigma-Aldrich, St. Louis, MI, USA) and 100 unit/mL penicillin/streptomycin (Gibco) at 37 °C in a 21% oxygen/5% CO2 incubator. 293-T cells were cultured in DMEM medium (ATCC) supplemented with 10% fetal bovine serum without penicillin/streptomycin. The details of the reagents used in this studied are summarized in Table S1. For the lipid raft formation inhibitor, MβCD was used at 10 mM in the medium. For AKT-mTOR pathway inhibition, LY294002 (50 μM) was used as a final concentration in the medium. The CPT1 inhibitor at different doses (37.5 and 75 μM) was used in this experiment. The product details are included in Table S1.

The cell lines used in our study have their own characteristics. LNCaP is a PCa adenocarcinoma cell line which is abundant with the androgen receptor (AR), sensitive to androgen deprivation therapy (ADT) and enzalutamide therapy. LNCaP/AR cell is LNCaP with AR overexpression. It is resistant to ADT but sensitive to enzalutamide. LNCaP/AR-shP53/shRB KD is an NE-like PCa cell line which is resistant to both ADT and enzalutamide. Both LNCaP/AR and LNCaP/AR-shP53/shRB KD cell lines are from Charles Sawyers’ Lab [18]. The C4-2 cell line is originated from the LNCaP cell line and is resistant to ADT. However, the C4-2 cell line is sensitive to enzalutamide, a second-generation hormone therapy drug for PCa. C4-2/MDVR cells are generated after six months of treatment with enzalutamide from Allen Gao’s lab. It is an adenocarcinoma cell line but with enzalutamide resistance characteristic. PC3 cell line is an NE-like PCa cell line with AR loss and resistance to enzalutamide. CWRR1 is an androgen resistant cell line that expresses the full-length androgen receptor, splice variant 7 AR and the luminal epithelia markers.

### 2.2. Cell Proliferation Analysis

To determine the effects of ELOVL5 on cell proliferation and growth curve, hemocytometer Cell Counting was used according to the instructions. Briefly, we cleaned the chamber and added 10 μL of the cells to the hemocytometer after harvest cells. Then, we placed the chamber in a microscope under a 10× objective. We counted the cells in the peripheral 4 squares (1 mm^2^) and calculated the average number. Duplicate samples were prepared and counted as an average.

### 2.3. CRISPR/Cas9-Mediated ELOVL5 Knockout

To establish ELOVL5 knockout stable cell lines, the CRISPR/Cas9 technology was utilized in the C4-2 cell line. The CRISPR for ELOVL5 was bought from GeneCopoeia (Rockville, MD, USA) of which information is summarized Table S1. Cells were selected by geneticin in the concentration of 500 μg/mL. Results were confirmed by Western blot and QPCR (Quantitative Polymerase Chain Reaction) analysis.

### 2.4. Lipid Raft Assay

The Lipid Raft Labeling Kit (Vybrant™ Alexa Fluor™ 555, V34404, Thermo Fisher, Waltham, MA, USA) was used in this research according to the manufacturer’s instructions. In brief, we added 2 µL of the 1 mg/mL cholera toxin subunit B (CT-B) conjugate stock solution to a final volume of 2 mL chilled complete growth medium. To get the stock solution, we added 100 µL of the 1× PBS to the vial with the Alexa Fluor dye, labeled CT-B (Component A), and gently dissolved the solid. After culturing for 30 min, we observed the cells under florescence microscope.

### 2.5. Lentivirus Package

The generated procedure follows the protocol described before [19,20]. 293FT cells were cultured in a 10 cm dish to 90% confluency. DNA mixture was prepared (1.5 mL OptiMem + 4.2 ug PMDL + 2 ug PREV + 2.8 ug VSVG + 5 ug plasmid of interest) and then mixed with Lipofectamine lipid. DNA/Lipofectamine mixture was added to each dish. After 24 h incubation, the old media was removed and 15 mL fresh collection media was added to each dish. After another 48 h incubation, we collected the supernatant which contained the virus and then filtered through 0.4 µm microfilter (VWR Sterile Syringe Filter, Monroeville, PA, USA) to collect the virus. 

### 2.6. RNA Interference

SiRNA targeting human ELOVL5 was used in this study with the details shown in Table S1. After 24 h, 30 pmol/L (picomoles per liter) SiELOVL5 or SiCtrl was transfected into cells using Lipofectamine RNAiMAX (Invitrogen, Carlsbad, CA, USA) in Opti-MEM reduced serum medium (Gibco) for 48−72 h according to the instructions.

### 2.7. Metabolite Extraction and Mass Spectrometry

As described previously [21], 10^5^ cells/well were plated in a 6-well plate. After being washed with pre-cold PBS and scraped in methanol that is −80 °C, the dry pellets were sent for further liquid chromatography mass spectrometry analysis (LC–MS). Data were normalized to cell numbers. LNCaP, LNCaP-AR, LNCaP/AR-shP53/shRB, and PC3 cell lines were used in this study. All of the experiments were conducted in biological triplicates.

### 2.8. MTS Analysis

The MTS assay (Biovision, San Francisco Bay, CA, USA) was employed to determine cell viability following the manufacturer’s protocol. Briefly, 1000/well cells were seeded in a 96-well plate. Ten μL/well MTS Reagent was added into each well and incubated for at least 1 h. Absorbance was measured at 490 nm using the SpectraMax M3 reader with SoftMax Pro 6 software (V5.3) for data acquisition and analysis. For cells proliferation assay, the results of each group were normalized to Day 0. For IC50 determination of CPT1-inhibitor, cells were treated by a series of concentration for 72 h. IC50 value was calculated by the GraphPad Prism software (San Diego, CA, USA). All assays were performed in triplicate.

### 2.9. Xenograft Mice Model

Nude (nu/nu) mice were bought from the Shanghai Laboratory Animal Center (SLAC, Shanghai, China). A total of 5 × 10^6^ of LNCaP or LNCaP-ELOVL5 OE cells were inoculated subcutaneously into six-week-old male nude mice. Tumor volume (mm^3^) = (length × height)/2. Enzalutamide was administered twice a week through intraperitoneal injection at a dose of 30 mg/kg body mass for 2 weeks. Four mice were used in each group. After the samples were collected, all of them are put into paraformaldehyde and embedded in paraffin wax for further IHC/HE staining.

### 2.10. Statistical Analysis

Statistical analysis was performed using Student’s *t* test by SPSS software (version 19.0; SPSS Inc., Chicago, IL, USA). *p* values are denoted as follows: * *p* < 0.05, ** *p* < 0.005, *** *p* < 0.001.

## 3. Results

### 3.1. Fatty Acid Elongation Is Upregulated by ADT Treatment and Elevated in NE-Like PCa Cells

**Figure 1:**
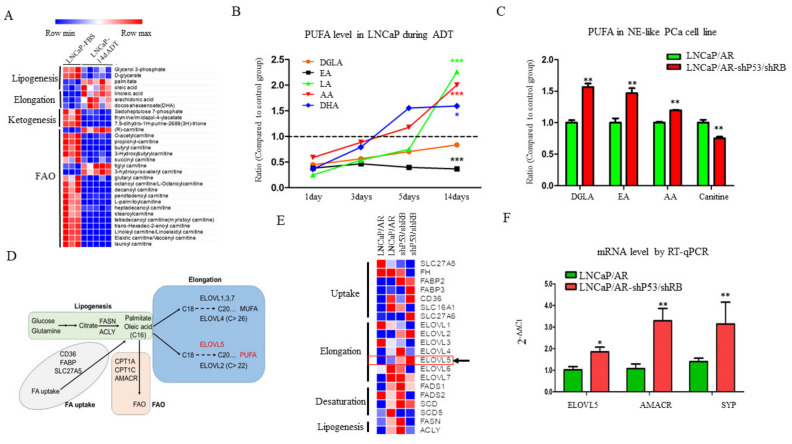
PUFA is upregulated upon ADT treatment and elevated in NE-like prostate cancer cells. (**A**). Heatmap of metabolites in control or 14-day ADT treated LNCaP cells. Long chain fatty acid concentration is upregulated significantly after ADT treatment. (**B**). Poly unsaturated fatty acid (PUFA) changes during the 14-day ADT treatment course in LNCaP cells. DGLA (Dihomo gamma linolenic acid), AA (arachidonic acid), LA (linoleic acid), DHA (docosahexaenoic acid), EA (eicosatetraenoic acid). (**C**). PUFA concentration in LNCaP/AR-shP53/shRB cells is elevated significantly (*p* < 0.01) compared to LNCaP/AR cells. (**D**). Schematic presentation for the fatty acid metabolic pathways that include lipogenesis, fatty acid uptake, fatty acid elongation and fatty acid oxidation (FAO). (**E**). Heatmap for genes related to fatty acid metabolism in the LNCaP/AR and LNCaP/AR-shP53/shRB cells. ELOVL5, the key enzyme for fatty acid elongation, is increased in the LNCaP/AR-shP53/shRB cell line. (**F**). ELOVL5 mRNA is increased significantly (*p* < 0.05) in mRNA level. AMACR and SYP function as positive controls. For all panels unless otherwise noted, mean ± SEM (error bars) is represented, and *p* values were calculated using t tests. N.S., not significant. * *p* < 0.05, ** *p* < 0.01, *** *p* < 0.001.

We have used metabolomics to profile the metabolites in androgen dependent prostate cancer cell line LNCaP with or without ADT treatment and characterized a glutaminse isoform switch mechanism to drive the therapy resistance of prostate cancer [20]. We further analyzed the lipid metabolites involved in lipogenesis, lipid elongation, ketogenesis and fatty acid β-oxidation (FAO), and observed that fatty acid synthesis and elongation, especially three PUFAs (polyunsaturated fatty acids) Linoleic acid (LA), Arachidonic acid (AA) and Docosahexaenoic acid (DHA), are significantly enhanced in the 14-day treated LNCaP cells (Figure 1A). Interestingly, the levels of the three PUFAs (LA, AA and DHA) in LNCaP cells were increased over the ADT treatment time (Figure 1B and Figure S1A), which is correlated with the appearance of NE (neuroendocrine)-like phenotype after ADT treatment [19,22,23,24,25], suggesting that PUFAs might be elevated with the ADT-induced NE development. To directly test whether PUFA level is increased in NE-like PCa cells, we profiled metabolites in LNCaP/AR and LNCaP/AR-shP53/shRB cells (representing adenocarcinoma PCa cells and NE-like PCa cells, respectively [18] (Figure S1B). The levels of PUFAs, such as DGLA, EA and AA, were significantly increased (Figure 1C), indicating that PUFAs are upregulated in NE-like PCa cells. PUFAs are synthesized by elongation and desaturation of C16 palmitate or oleic acid mediated by a series of fatty acid elongates and desaturases (Figure 1D). The analyses of RNA-seq data of LNCaP/AR and LNCaP/AR-shp53/shRB [18] revealed that ELOVL5 was upregulated in NE-like enzalutamide resistant LNCaP/AR-shp53/shRB cells compared to LNCaP/AR cells (Figure 1E), which was further confirmed by RT-qPCR (Figure 1F) and the expression pattern in different cancer tissues (Figure S1C). Thus, ADT treatment upregulates the fatty acid elongation and ELOLV5, the critical regulator of PUFA, is overexpressed in NE-like PCa cells compared to adenocarcinoma PCa cells.

### 3.2. Fatty Acid Oxidation (FAO) Is Downregulated in NE-Like PCa Cells

**Figure 2:**
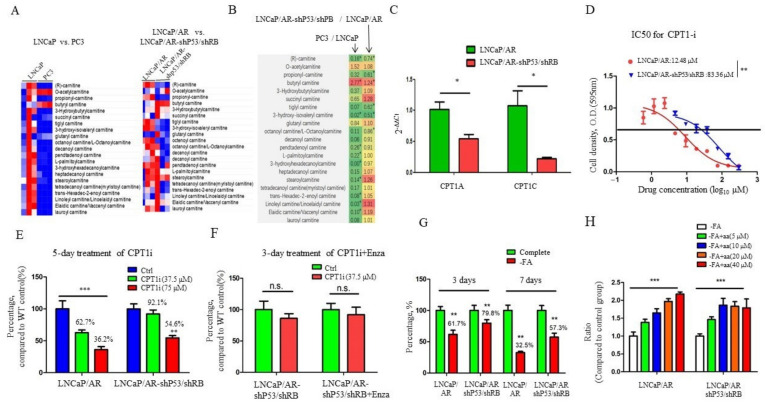
Fatty acid oxidation (FAO) is downregulated in NE-like prostate cancer cells. (**A**). Heatmap for fatty acyl-carnitines in NE-like PCa cell lines (PC3 and LNCaP/AR-shP53/shRB cells) versus adenocarcinoma PCa cells (LNCaP and LNCaP/AR). Fatty acyl-carnitines are mostly decreased in NE-like PCa cells compared to the adenocarcinoma cells. (**B**). Fatty acyl-carnitine changing folds in PC3/LNCaP and LNCaP/AR-shP53/shRB/LNCaP/AR cells. Green: ratio upregulated, red: ratio downregulated. (**C**). CPT1A and CPT1C mRNA levels in LNCaP/AR and LNCaP/AR-shP53/shRB cells. Both CPT1 isoforms are decreased in LNCaP/AR-shP53/shRB cells significantly. (**D**). IC50 of CPT1 inhibitor in different cell lines. NE-like PCa cell line LNCaP/AR-shP53/shRB displays decreased IC50 value of CPT1 inhibitor. (**E**). Cell number changes in LNCaP/AR and LNCaP/AR-shP53/shRB cells after 5-day treatment of CPT1i. (**F**). Cell number changes in LNCaP/AR-shP53/shRB cells after 3-day treatment of enzalutamide (20 uM) combined with CPT1i. (**G**). Cell number changes in LNCaP/AR and LNCaP/AR-shP53/shRB cultured with complete media or FA depleted medium for 3 days or 7 days. (**H**). Arachidonic acid (AA)’s effect on the proliferation of LNCaP/AR and LNCaP/AR-shP53/shRB cells. LNCaP/AR-shP53/shRB cells present more sensitivity to AA supplement. For all panels unless otherwise noted, mean ± SEM (error bars) is represented, and *p* values were calculated using t tests. N.S., not significant. * *p* < 0.05, ** *p* < 0.01, *** *p* < 0.001.

Fatty acid oxidation, also known as β-oxidation, is a major metabolic pathway by which fatty acid is oxidized to release energy by removing two carbon unit acetyl-coA and carnitine plays a critical role in the β-oxidation by transporting fatty acyl-CoA across the mitochondria membrane [26]. To better understand the fatty acid metabolism and its oxidation during the NE-like enzalutamide resistant PCa development, we compared the fatty acid oxidation (in the form of fatty acyl-carnitine) in two pairs of PCa cells: LNCaP vs. PC3 (representing adenocacinoma vs. small cell neuroencrine PCa cells) and LNCaP/AR vs. LNCaP/AR-shp53/shRB. As shown in Figure 2A, except for O-acetylcarnitine and butyrylcarnitine, most fatty acylcarnitine were significantly reduced in their levels in the PC3 and LNCaP/AR-shp53shRB cells compared to LNCaP and LNCaP/AR, respectively (Figure 2A,B), indicating that FAO is downregulated in NE-like PCa cells. CPT1 is the rate-limiting enzyme that mediates β-oxidation [7]. Both isoforms of CPT1, CPT1A and CPT1C are significantly reduced in their mRNA levels in LNCaP/AR-shp53/shRB cells (Figure 2C). Furthermore, LNCaP/AR-shp53/shRB PCa cells were more resistant to CPT1 inhibitor (etomoxir) than LNCaP/AR cells as shown by the IC50 values of 83.36 μM vs. 12.48 μM and cell proliferation at different doses of etomoxir (37.5 and 75 μM, Figure 2D,E). However, the combined use of CPT1i with enzalutamide did not alter the enzalutamide sensitivity for LNCaP/AR-shp53/shRB PCa cells (Figure 2F). All these findings are corroborated with the downregulation of FAO in NE-like PCa cells and suggest that FAO might not be involved in enzalutamide resistance.

In addition to the de novo biosynthesis, fatty acid can be uptaken by cells from extracellular media. We have demonstrated that prostate cancer cells are less glycolytic for both adenocarcinoma and NE-like cells [20]. To examine the fatty acid dependence of prostate cancer cells, we cultured LNCaP/AR and LNCaP/AR-shp53/shRB cells in media depleted with fatty acid. As shown in Figure 2G, LNCaP/AR-shp53/shRB PCa cells are less sensitive to fatty acid depletion than LNCaP/AR cells (the cell survival rate, 79.8% vs. 61.7% in 3-day treatment and 57.3% vs. 32.5% in 7-day treatment, all *p* values < 0.01), suggesting that NE-like PCa cells may use de novo synthesized fatty acid to generate energy for proliferation. Interestingly, when replenished with AA (5–40 μM) in the fatty acid-depleted media, both cell lines showed the rescue of cell proliferation, but LNCaP/AR-shp53/shRB cells showed a more quicker rescue response (Figure 2H). Taken together, FAO is reduced in NE-like PCa cells and more fatty acids might be involved in the elongation process.

### 3.3. ELOVL5 Mediates Enzalutamide Resistance in NE-Like PCa Cells

**Figure 3:**
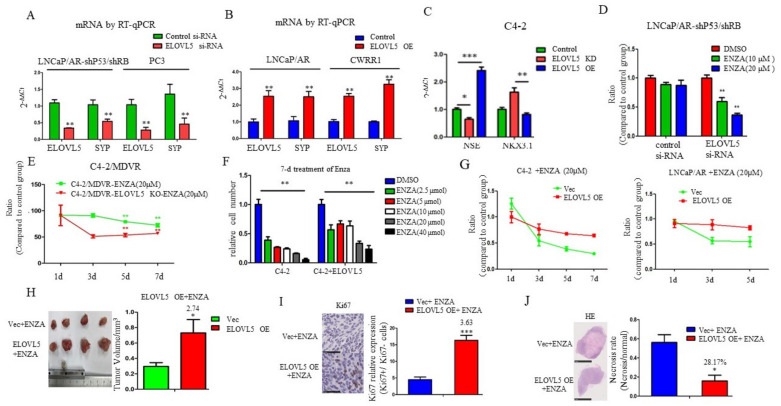
ELOVL5 regulates enzalutamide sensitivity and resistance of prostate cancer cells. (**A**). Changes of ELOVL5 and SYP mRNA level in NE-like PCa cells (LNCaP/AR-shP53/shRB and PC3) with the ELOVL5 knockdown by siRNA. (**B**). Changes of ELOLV5 and SYP mRNA level in adenocarcinoma PCa cells (LNCaP/AR and CWRR1) with the ELOVL5 overexpression. (**C**). Changes of NSE and NKX3.1 mRNA levels in C4-2 cells with ELOVL5 knockout by CRISPR-Cas9 or ELOVL5 overexpression. (**D**). Survival rate of LNCaP/AR-shP53/shRB with ELOVL5 knockdown by siRNA after 7-day enzalutamide treatment at different doses. (**E**). Cell growth curves of C4-2/MDVR and C4-2/MDVR-ELOVL5 knockout by CRISPR-Cas9 with enzalutamide treatment. (**F**). Cell numbers in C4-2/control and C4-2/ELOVL5 overexpressing cells treated with enzalutamide at different concentrations. (**G**). Cell growth curves of different cell lines (C4-2 and LNCaP-AR) with ELOVL5 overexpression treated with enzalutamide. (**H**). Tumor sizes of LNCaP/Control and LNCaP/ELOVL5 overexpressing xenograft tumors treated with enzalutamide, fold change: 2. 74. (**I**). Ki67 positive staining of LNCaP/Control and LNCaP/ELOVL5 xenograft tumors treated with enzalutamide, fold change: 3.36. (**J**). Necrosis staining of LNCaP/Control and LNCaP/ELOVL5 xenograft tumors treated with enzalutamide, ratio: 28.17%. For all panels unless otherwise noted, mean ± SEM (error bars) is represented, and *p* values were calculated using *t* tests. N.S., not significant. * *p* < 0.05, ** *p* < 0.01, *** *p* < 0.001.

Treatment-induced neuroendocrine development for PCa is one of the enzalutamide resistance mechanisms [18]. Since PUFA is elevated and ELOVL5 expression is upregulated in NE-like PCa cells (Figure 1), we wanted to know whether ELOVL5-mediated fatty acid elongation contributes to enzalutamide resistance. Knockdown of ELOVL5 by siRNA in two NE-like PCa cell lines, LNCaP/AR-shp53/shRB and PC3, led to the downregulation of neuroendocrine markers SYP (Figure 3A) whereas overexpression of ELOVL5 in LNCaP/AR and CWRR1 cells resulted in the upregulation of SYP (Figure 3B), suggesting that ELOVL5 may be involved in NE development. This is further confirmed by the observation that modulating ELOVL5 expression in C4-2 by either CRISPR-Cas9 knocking out or overexpressing led to the opposite change of NSE and NKX3.1 expression, two markers representing neuroendocrine PCa and luminal PCa cells, respectively (Figure 3C). Further, knockdown of ELOVL5 by siRNA in LNCaP/AR-shp53/shRB cells or CRISPR-Cas9 in C4-2/MDVR cells, both enzalutamide resistant PCa cell lines, increased their enzalutamide sensitivity (Figure 3D,E). On the other hand, overexpression of ELOVL5 in C4-2 or LNCaP/AR cells conferred them the enzalutamide resistance (Figure 3F,G). In the xenograft model, ELOVL5 overexpressing LNCaP xenograft tumors were significantly larger than the control xenograft tumors after treatment with enzalutamide (Figure 3H). IHC staining of the xenograft tumors showed that ELOVL5 overexpression led to more proliferation and less necrosis in the presence of enzalutamide treatment as shown by Ki67 and H& E staining (Figure 3I,J). All these in vitro and in vivo results indicate that ELOLV5 overexpression contributes to the enzalutamide resistance of PCa.

### 3.4. Lipid Raft Associated AKT-mTOR Pathway Is Involved in ELOVL5 Induced Enzalutamide Resistance

**Figure 4:**
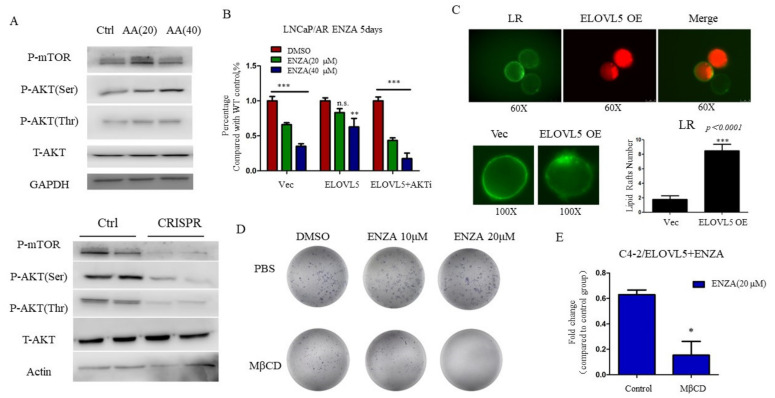
Lipid raft derived AKT-mTOR pathway mediates ELOVL5 induced enzalutamide resistance. (**A**). AKT-mTOR pathway is upregulated in AA treated C4-2 cells whereas is down-regulated in ELOVL5 KO C4-2 cells. Complete blots are available in Figures S3 and S4. (**B**). AKT inhibitor increases the enzalutamide sensitivity of LNCaP/AR-ELOVL5 cells. (**C**). Number of lipid raft is elevated after ELOVL5 overexpression in C4-2 cells. (**D**). Lipid raft inhibitor (MβCD, 10 mM) diminishes the enzalutamide resistance of ELOVL5 overexpressing C4-2 cells. (**E**). Fold change of cell number between control and MβCD treated C4-2/ELOVL5 expressing cells treated with enzalutamide. For all panels unless otherwise noted, mean ± SEM (error bars) is represented, and *p* values were calculated using t tests. N.S., not significant. * *p* < 0.05, ** *p* < 0.01, *** *p* < 0.001.

The AKT-mTOR signaling pathway has been shown to regulate enzalutamide resistance of PCa and this pathway is also reported to be regulated by fatty acid metabolism [13,27]. To examine whether the AKT-mTOR pathway is involved in ELOVL5-induced enzalutamide resistance, we supplemented AA (24 h treatment, which is synthesized and elongated by ELOVL5) in the culture medium for C4-2 cells, and found that the AKT-mTOR pathway was elevated after AA treatment (Figure 4A). Knocking out ELOLV5 by CRISPR-Cas9 in C4-2 cells resulted in decreased AKT-mTOR signaling (Figure 4A). To directly confirm the link between ELOLV5 and AKT-mTOR signaling, the AKT inhibitor (LY294002) was used to treat ELOVL5 expressing LNCaP/AR cells and was shown to enhance the enzalutamide sensitivity of ELOVL5-expressing LNCaP/AR cells (Figure 4B). Lipid raft has been reported as an important membrane microdomain for AKT activation [28]. The formation of lipid rafts were measured by lipid raft assay kit (Vybrant™ Alexa Fluor™ 555, V34404) and the numbers of lipid rafts were increased in ELOVL5 expressing cells (Figure 4C, *p* < 0.0001). Interestingly, the lipid raft inhibitor MβCD was shown to confer the C4-2/ELOVL5 cells the sensitivity to enzalutamide (Figure 4D) and abrogated the ELOVL5-induced enzalutamide resistance in C4-2 cells (Figure 4E). Taken together, these findings support that the lipid rafts associated AKT-mTOR pathway plays an important role in ELOVL5 induced enzalutamide resistance in NE-like PCa cells.

## 4. Discussion

**Figure 5:**
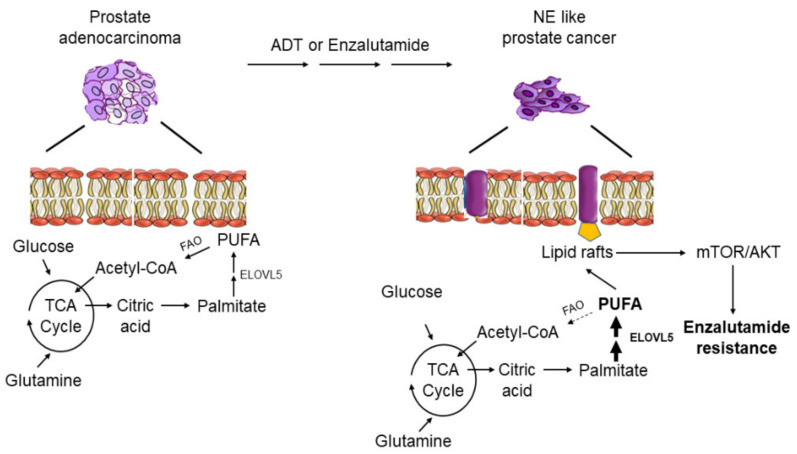
A working model of ELOVL5 induced enzalutamide resistance in NE-like PCa cells.

In this study, we identified the fatty acid elongation enzyme ELOVL5 as an important player in regulating the enzalutamide resistance of prostate cancer. We demonstrated that ELOVL5 was overexpressed in the NE-like PCa cells that mediates the elongation of fatty acid, resulting in the upregulation of PUFA in NE-like PCa cells. The elevated PUFA promotes more lipid raft clusters in the NE-like PCa cell membrane that facilitates the activation of AKT-mTOR signaling and therefore contributes to the enzalutamide resistance (Figure 5). These findings implicate that ELOLV5-mediated PUFA elongation may be a novel target for the treatment of enzalutamide resistant NE-like PCa.

Fatty acid metabolism includes de novo lipogenesis, fatty acid elongation, β-oxidation and fatty acid uptake (Figure 1C). De novo lipogenesis has been shown to be enhanced in prostate cancer, especially in more advanced prostate cancer [29]. Fatty acid synthase (FASN), one of the most important enzymes involved in fatty acid biosynthesis, is consistently overexpressed in prostate cancer tissues compared with the adjacent normal tissue [30], and the FASN inhibitor has been shown to suppress the progression of cancers, including prostate cancer [29,31]. To meet the energy demands in cells, the uptake and β-oxidation of fatty acids are always coordinately regulated in order to ensure an adequate supply for mitochondrial β-oxidation [26,32]. The uptake of fatty acid by specialized transporters provides an important compensatory mechanism for cancer cells to maintain their lipid demands and also provide sufficient ATP to fuel the metastatic cascade through β-oxidation [32,33]. The AMP-activated protein kinase (AMPK)-CPT1 signaling pathway has been shown to play an important role in the β-oxidation [8]. We found that β-oxidation and its regulator CPT1 A/C mRNA levels were both downregulated in NE-like PCa cells, suggesting that NE-like PCa cells are less dependent on the fatty acid uptake and β-oxidation, which is further supported by the observation that CPT1 inhibitor did not have effects on the proliferation of NE-like PCa cells. These findings also suggest that NE-like PCa cells may tend to accumulate more PUFAs. 

PUFAs are synthesized originally from palmitate or oleic acid through elongation by elongases and desaturation by stearoyl-CoA desaturases. The ELOVL family (ELOVL1–7) is critical for the fatty acid elongation [9,16]. ELOVL2 and ELOVL5 are mainly responsible for the PUFA production (Figure 1C) and ELOVL5 is most highly expressed in prostate tissue (Figure S1C). Consistent with the PUFA elevation, we also found that ELOVL5 was overexpressed in enzalutamide-resistant PCa cells and modulating ELOVL5 expression did alter the enzalutamide resistance of NE-like PCa cells as well as the neuroendocrine characteristics. All these findings implicate ELOVL5 as a potential regulator of enzalutamide resistance of PCa.

There are different families of PUFA according to the location of double bonds. The n-3 family of PUFAs includes alpha-linolenic acid (ALA), eicosapentaenoic acid (EPA) and docosahexaenoic acid (DHA). The n-6 family includes linolenic acid (LA) as well as arachidonic acid (AA). Though different PUFA has its own effects on cell survival, AA (AA, 20:4 n-6) is a precursor for pro-inflammatory lipid mediators [34]. AA has also been shown to increase glucose receptor levels in the cell membranes, which potentially might increase de novo lipogenesis [9,31]. More importantly, PUFAs constitute the structure of the cellular lipid membrane. Unsaturated fatty acids are flexible and easier to bend, which can create a more liquid cell membrane, and therefore it is much easier for cells to communicate with the microenvironment [12,35]. Furthermore, PUFAs also contribute to the formation of lipid rafts and facilitates the activation of cellular signaling pathways, as shown by a study wherein lipid raft inhibition led to the suppression of lipid biosynthesis and AKT-mTOR pathways [12]. Thus, our study here demonstrated that the elevated PUFAs and ELOVL5 expression in NE-like PCa cells promotes that lipid raft formation and facilitates the activation of the AKT-mTOR pathway, resulting in the enzalutamide resistance of NE-like PCa. Although there is still no ELOVL5 inhibitor available, our study may strongly suggest ELOVL5 as a novel target for treating the enzalutamide-resistant NE-like prostate cancer. Finally, peroxidation of PUFAs by lipoxygenases has been shown to drive ferroptosis, which may herald a new treatment approach for enzalutamide resistant PCa by inducing ferroptosis [36].

Our study used multiple model systems, but there are still several limitations. Firstly, the xenograft tumor model is unable to mimic the prostate cancer tumor environment, and therefore it is still unknown how fatty acid affects the tumor environment. Secondly, there is still no specific inhibitors for ELOVL5 and it is thus difficult to pharmacologically target the PUFA elongation and examine their effects on enzalutamide resistance; however, our work warrants further investigation and to develop the ELOVL5 specific inhibitor for the treatment of NEPC. Finally, more clinical samples are needed to test the ELOVL5 effects on the enzalutamide resistance and NE lineage switch of prostate cancer.

## 5. Conclusions

ELOVL5-mediated PUFA elongation enhances the lipid raft-associated AKT-mTOR signaling activation, which is critical in enzalutamide resistance of prostate cancer.

## Data Availability

All data analyzed are included in this article and additional information is available upon request.

## Supplementary Materials

The following are available online at https://www.mdpi.com/article/10.3390/cancers13163957/s1, Figure S1: Fatty acid elongation related to Figure 1, Figure S2: ELOVL5 overexpressed and knocked down cell lines, Figure S3: Complete Blots of Figure 4, Figure S4: Complete blots of Figure S1 and S2, Table S1: Resource of the reagent.
