# Supplementary material for: ELOVL5-Mediated Long Chain Fatty Acid Elongation Contributes to Enzalutamide Resistance of Prostate Cancer
Source: Cancers (Basel). 2021 Aug 5;13(16):3957. doi: 10.3390/cancers13163957 (PMC8391805; doi:10.3390/cancers13163957)
Supplement: Supplementary file 1 [file cancers-13-03957-s001.zip › cancers-1296935-supplementary.pdf]

cancers

MDPI

Supplementary Materials: ELOVL5-Mediated Long Chain Fatty Acid Elongation Contributes to Enzalutamide Resistance of Prostate Cancer

Huan Xu [1,2,†], Sangsang Li [3,†], Yi Sun [4,5,†], Lingfan Xu [6], Xin Hong [3], Zhong Wang [1,*] and Hailiang Hu [3,*]

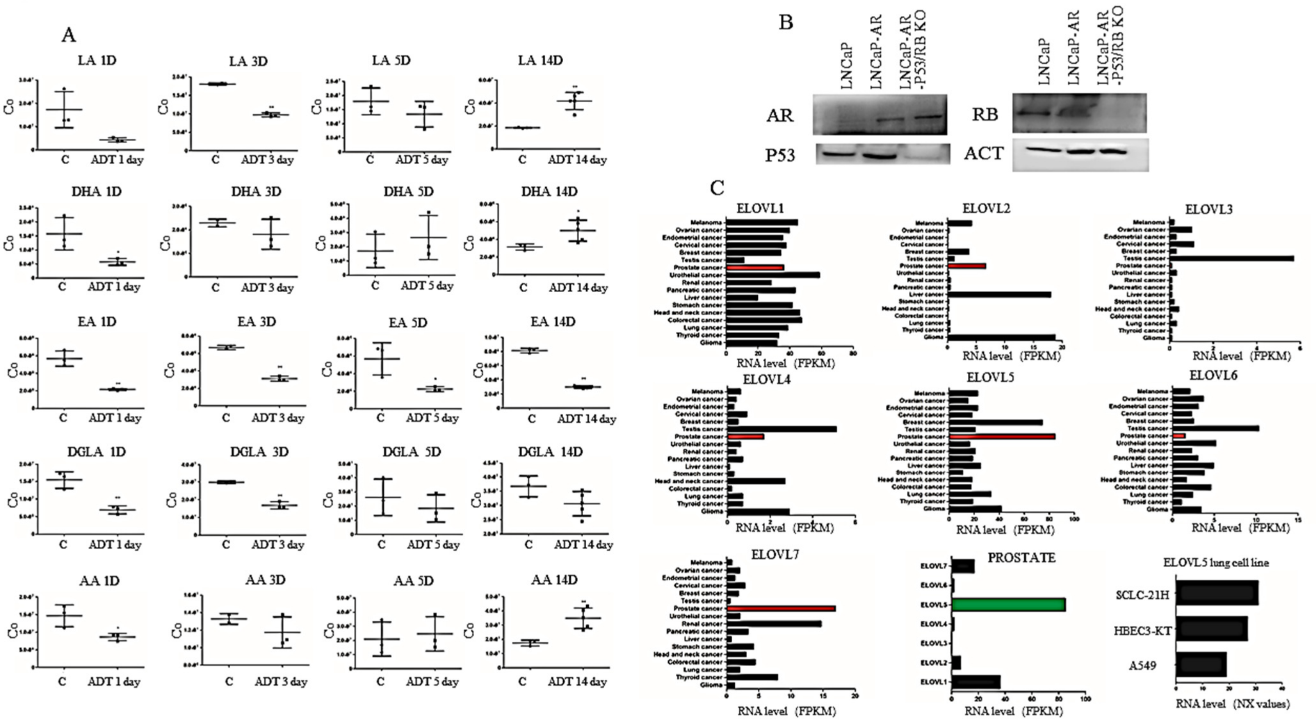

Figure S1. Fatty acid elongation related to Figure 1. (**A**) Different PUFA levels with the ADT treatment course. (**B**) AR, P53, RB expression in LNCaP, LNCaP/AR and LNCaP/AR-shP53/shRB cell lines. (**C**) ELOVL family expression in different tissues according to TCGA dataset. For all panels unless otherwise noted, mean ± SEM (error bars) is represented, and p values were calculated using t tests. N.S., not significant. * $p < 0.05$, ** $p < 0.01$, *** $p < 0.001$.

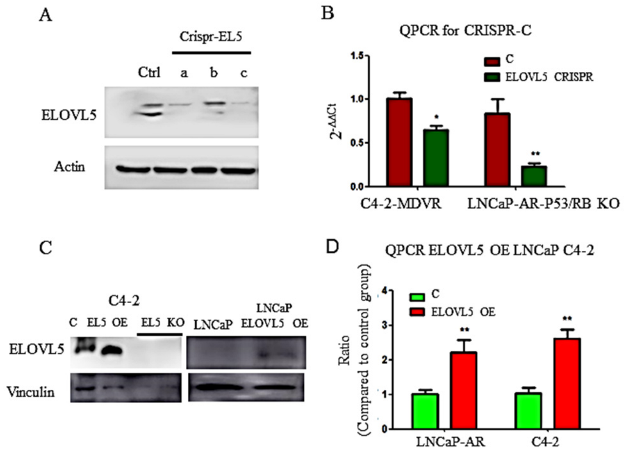

Figure S2. ELOVL5 overexpressed and knocked down cell lines. (**A**)ELOVL5 protein level after CRISPR-Cas9 knocked out in C4-2 cells. (**B**) ELOVL5 mRNA levels after CRISPR-Cas9 knocked out in C4-2/MDVR and LNCaP/AR-cells. (**C**) ELOVL5 protein level in different cell lines with ELOVL5 overexpression. (**D**) ELOVL5 mRNA levels with ELOVL5 overexpression

in LNCaP/AR and C4-2 cell lines. For all panels unless otherwise noted, mean ± SEM (error bars) is represented, and p values were calculated using *t* tests. N.S., not significant. * $p < 0.05$, ** $p < 0.01$, *** $p < 0.001$.

Table S1. Resource of the reagent

Reagent or Resource<br>Chemicals, Peptides, and Recombinant Proteins	Source	Identifier
RPMI-1640 medium	ATCC	30-2001
DMEM medium	ATCC	30-2002
Fetal Bovine Serum (FBS)	Corning	35-010
Geneticin	Thermo Fisher	10131027
Penicillin/streptomycin	Gibco	15140-163
Lipo-3000	Thermo Fisher	L3000001
TRIzol	Thermo Fisher	15596-018
SiRNA ELOVL5	Santa Cruz	Sc-62270
CRISPR-ELOVL5	GeneCopoeia	HCP308677-CG12-3-B
ELOVL5 overexpression plasmid	GeneCopoeia	EX-A1818-M03-GS
CPT1 inhibitor Etomoxir	Thermo Fisher	ST1326
Palmate	Sigma-Aldrich	P9767
Oleic acid	Sigma-Aldrich	O1008
Delipidized Fetal Bovine Serum	Sigma-Aldrich	9048
Arachidonic acid	Sigma-Aldrich	10931
LY294002	CST	9901
MβCD	Aladdin	M102038
**Antibodies**	**Source**	**Identifier**
ELOVL5 Antibody	Abcam	ab205535
p-mTOR (Ser 2448) Antibody	CST	5536
t-AKT Antibody	CST	9272
p-AKT (Ser 473) Antibody	CST	4060
p-AKT (Thr 308) Antibody	CST	13038
CgA(Chromogranin A) Antibody	CST	60893
GAPDH Antibody	CST	5174
beta-Actin	CST	4970S

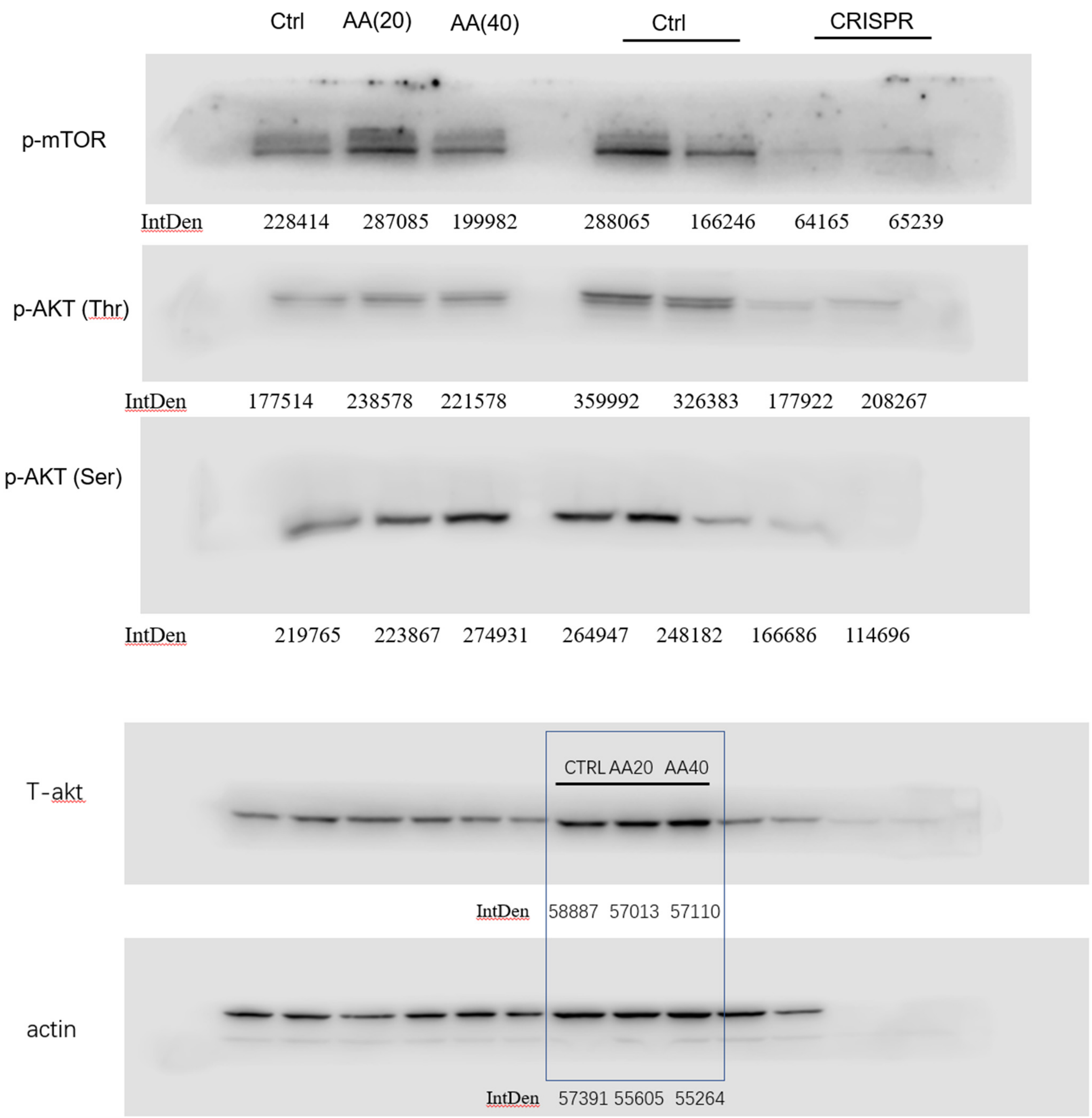
Ctrl
AA(20)
AA(40)
Ctrl
CRISPR
p-mTOR
IntDen 228414 287085 199982 288065 166246 64165 65239
p-AKT (Thr)
IntDen 177514 238578 221578 359992 326383 177922 208267
p-AKT (Ser)
IntDen 219765 223867 274931 264947 248182 166686 114696
T-akt
CTRL AA20 AA40
IntDen 58887 57013 57110
actin
IntDen 57391 55605 55264

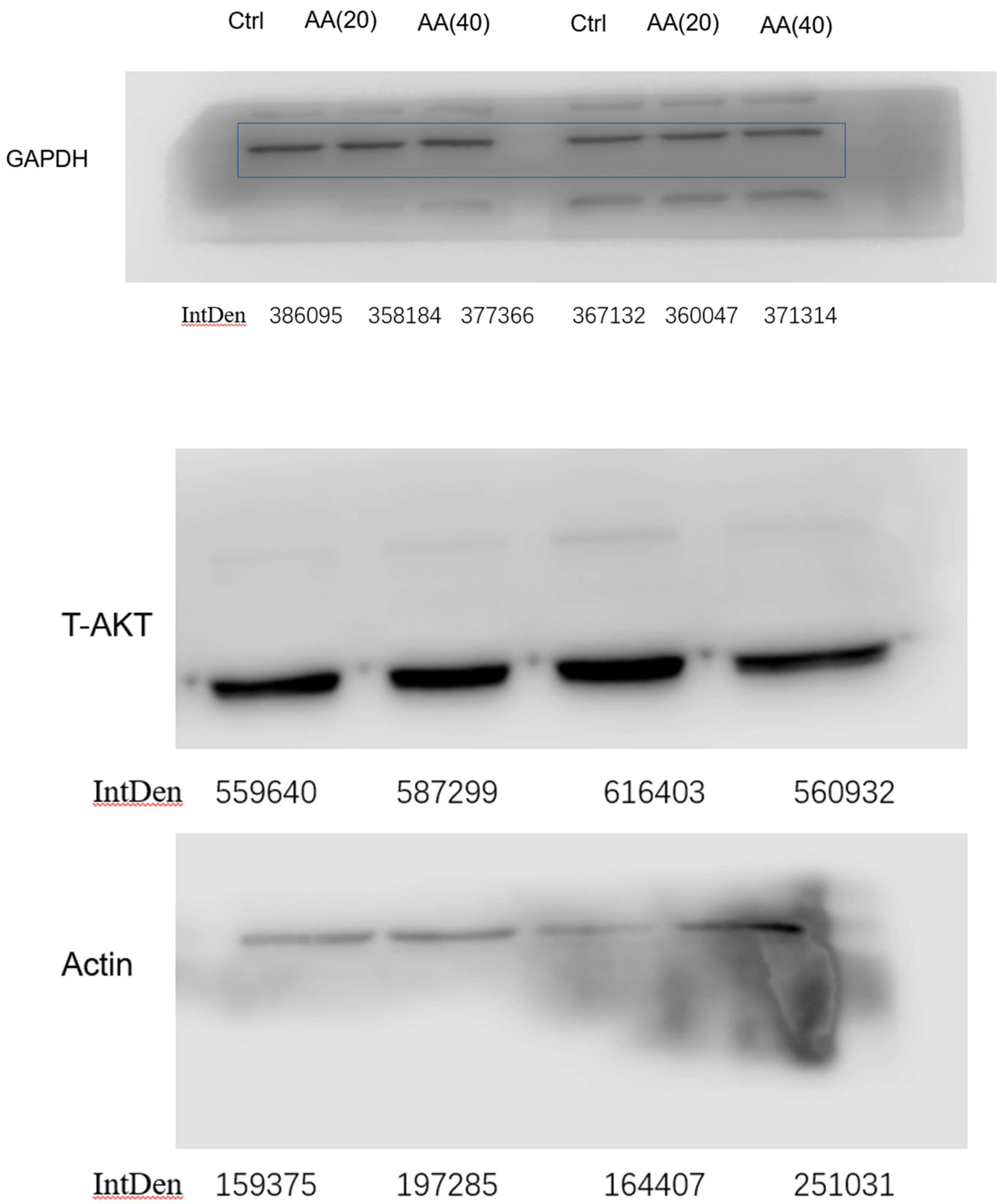

Figure S3. Complete Blots of Figure 4.

p53

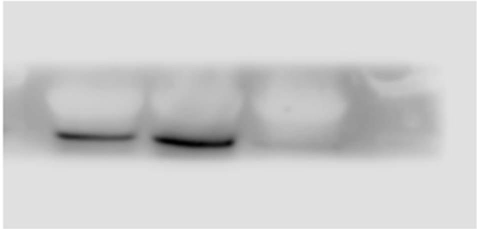

RB1

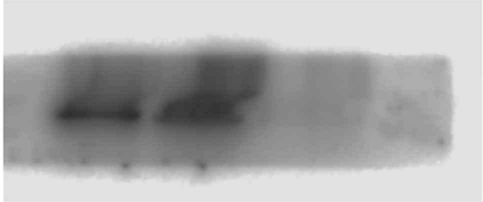

AR

T-akt
(not used)

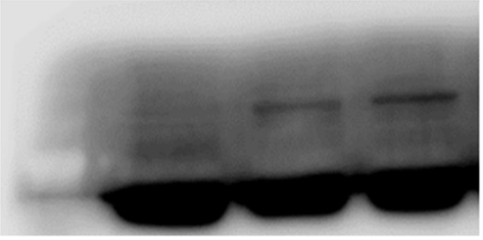

actin

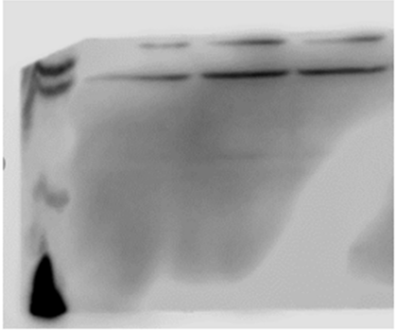

20190829

ELOVL5

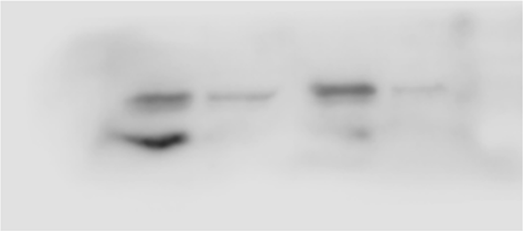

Actin

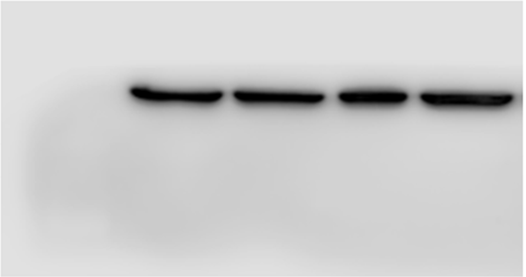

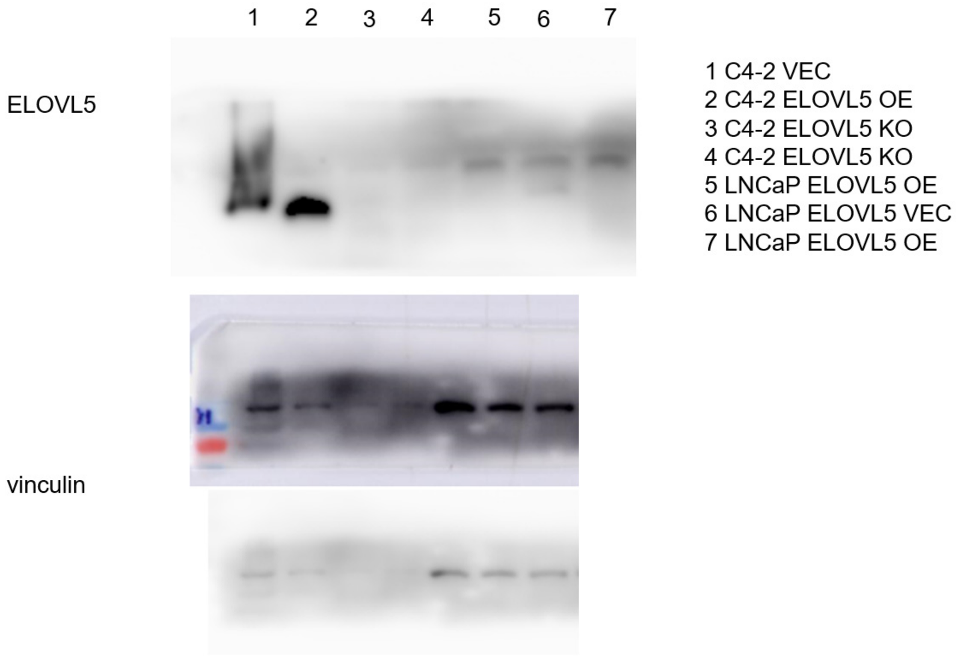

Figure S4. Complete blots of Figure S1 and S2.
